# Fertility after recurrent miscarriages: results of an observational cohort study

**DOI:** 10.1007/s00404-017-4532-4

**Published:** 2017-10-16

**Authors:** Christiane Kling, Jürgen Hedderich, Dieter Kabelitz

**Affiliations:** 1 Institute of Immunology, University Hospital Schleswig-Holstein, Campus Kiel, Arnold-Heller-Str. 3 Haus 17, 24105 Kiel, Germany; 2 Institute of Medical Statistics and Informatics, University Hospital Schleswig-Holstein, Campus Kiel, Arnold-Heller-Str. 3 Haus 31, 24105 Kiel, Germany; 3 Present Address: MVZ Dr. Fenner and Colleagues, Bergstr. 14, 20095 Hamburg, Germany

**Keywords:** Artificial reproductive technologies (ART), Idiopathic, Immunotherapy, Late miscarriage, Preclinical loss, Spontaneous conception, Tubal incompetence

## Abstract

**Purpose:**

Recurrent pregnancy losses (RPL) are considered a pathological condition associated with heterogeneous laboratory and clinical findings, and are also linked to subfertility. We attempt to rank parameters derived from past history and diagnostic results with regard to the prognosis.

**Methods:**

Observational trial on 719 consecutive couples who were referred to a tertiary immunological care centre (2006–2014) after three or more primary miscarriages. Information on past obstetric history and diagnostic procedures at baseline were correlated with cumulative pregnancy and delivery rates using Kaplan–Meier estimation, logistic regression and multivariate analysis.

**Results:**

At baseline, median female age was 34.1 years, waiting time 3 years (1–17), number of preceding miscarriages 3 (3–9), 147 women (20.4%) had conceived at least once in ART or AIH cycles. After a median follow-up of 33.7 (1.7–123.0) months, 5-year pregnancy and delivery rates were 86.1 and 64.5%. Female age (< 35 years), waiting time (< 3 years) until baseline, tubal competence, and male factor fertility significantly correlated with favourable outcome (*p* < 0.001), while body mass index (> 29 kg/m^2^), number of preceding miscarriages (> 4), late miscarriages, preclinical losses and smoking revealed non-significant negative trends. Mode of conception until baseline (spontaneously or ART/AIH) and classification into idiopathic and non-idiopathic RPL showed no prognostic relevance.

**Conclusion:**

Although in general, chances to conceive a child are retained after three or more miscarriages, factors related to subfertility of both partners have an important impact on the outcome. Therefore, prolonged time to pregnancy (> 6–9 months) should result in preventive gynaecological care from the first miscarriage on, so that fertility can be preserved as best as possible.

## Introduction

Recurrent pregnancy losses (RPL) are a distressful experience which affects about 1% of couples who attempt to conceive. In the 1970s, RPL has been defined as a maternal condition of having more than two consecutive losses up to the 20th gestational week [1].

With some variation concerning inclusion criteria, (e.g., late miscarriages over 20 weeks, couples with two miscarriages and with preclinical losses) this view gave rise to a concept to define RPL as a distinct disease. Since then, gynaecological guidelines are based on this concept [2–6]. They propose a diagnostic work-up of female parameters as well as cytogenetic aberrations of both partners in order to identify hormonal, genetic, uterine, haemostaseological, immunological, or other pathologies. These serve as an explanation for the condition and allow for therapeutic options in the following pregnancy. In about 50–75% of all cases, associated parental factors cannot be identified [6]. These idiopathic RPL were interpreted as immunological rejection of the embryo and foetus, and various immunotherapies were discussed as therapeutic options since the 1980s [7, 8].

At the same time, it is well-known that 80–90% of losses occur in the first trimester of pregnancy [9]. At least 50% of these clinical early miscarriages are cytogenetically abnormal, and in euploid pregnancy, gross embryonic malformations considerably contribute to early loss [10–12]. Experience derived from artificial reproductive technologies (ART) has shown that in the preimplantation phase, cytogenetic abnormalities and mosaicism of the embryo are frequent and related to maternal age [13]. In couples who had repeated miscarriages or recurrent implantation failure after in vitro fertilisation (IVF), the proportion of chromosomal disturbances was shown to be accentuated [14]. Thus, apparently during the 1st week of development the embryos undergo a wasteful process of self-selection which slows down, but is not completed at implantation, leading to the risk of miscarriage. According to this concept, a single loss may occur by chance, but repeated losses may indicate a suboptimal embryonic capacity to develop in an individual partnership. Epidemiological data have supported this view by showing that delivery rates after RPL can be excellent but decline with maternal age and number of preceding miscarriages [15–18].

Thus, the two concepts somewhat compete with each other [19, 20], and it is not clear, whether embryonic “quality” or surrounding processes in the maternal endometrium dominate the prognosis of an individual couple.

From the 1980s on, couples were referred to our tertiary care immunological outpatients department after three or more consecutive miscarriages by gynaecologic practitioners, geneticists, and reproductive medical centres in Germany. Lymphocyte immunotherapy (LIT) has been considered an optional treatment in selected cases of primary idiopathic recurrent miscarriages, as defined by the gynaecological guidelines. Therefore, most of the referred couples had undergone a diagnostic work-up accordingly in the referring centres.

The design of the study was not suitable to evaluate the therapeutic efficacy of LIT. Indirect evidence derived from our present data, however, suggests that this and other forms of immunotherapy may be inadequate to treat idiopathic recurrent pregnancy losses. Here we propose a different concept, as outlined below (see “Discussion”).

To generate information on general outcome, a preliminary observational study on 2-year pregnancy and delivery rates was conducted on couples referred in 1996–2003 after spontaneously conceived miscarriages [21]. The results suggested that RPL is linked to subfertility, with secondary sterility being an associated feature. This prospective follow-up study on couples referred consecutively in 2006–2014 gave us the chance not only to corroborate our previous results on outcome in a larger cohort but also to weigh the impact of diagnostic results and of subfertility. We included patients who had already started with ART or AIH at baseline and those who had experienced preclinical losses and non-idiopathic recurrent miscarriages.

Although the study is prospective in terms of baseline information, the design is not appropriate for examining the value of therapeutic interventions (e.g., LIT or ART/AIH during the observational period) because this would have required a randomized controlled approach.

## Methods

### Study group and design (Fig. 1)

LIT was introduced in our tertiary care outpatients department in the 1980s, and the method is approved by the local health authority. Diagnostic evaluation and treatment have mostly been covered by national health insurances, thus reference was not related to income. Patients were referred from various parts of the country, and 259 centres (including 84 of 128 IVF centres registered in the German IVF registry) contributed to the study. Criteria for recommending LIT have been outlined elsewhere [22, 23].

**Fig. 1:**
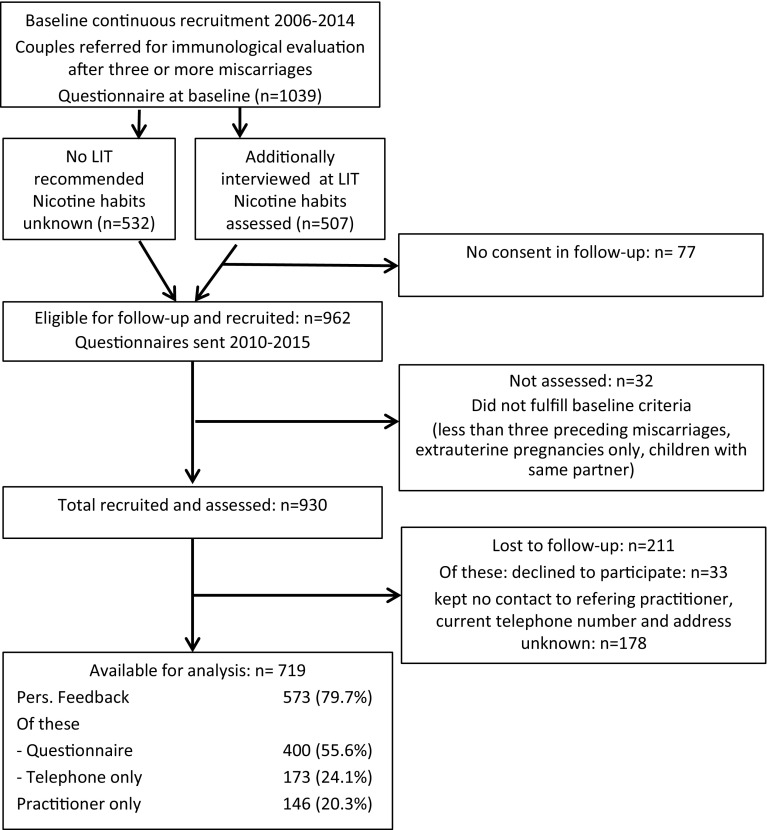
Follow-up flow diagram

The observational cohort study comprised 1039 couples who were consecutively referred from 2006 to 2014 and at primary assessment fulfilled the following criteria: three or more miscarriages within the 5th to 23rd gestational week after spontaneous conception, hormonal stimulation, insemination with the husband’s sperm (AIH) or in vitro fertilisation (IVF) with or without intracellular insemination with the partner’s sperm (ICSI), no deliveries in the same partnership. Diagnostic work-up by the referring practitioners partly adhered to the contemporary guidelines for diagnosis and treatment of recurrent miscarriages and comprised pelvic ultrasound, thyroid stimulating hormone (TSH), hysteroscopy, pelviscopy, cytogenetic evaluation of both partners, evaluation for haemostaseological abnormalities, and antiphospholipid syndrome. The referring practitioners indicated the results as well as details concerning the past miscarriages (gestational age, detection of embryonic vital signs, embryonic aneuploidy where tested) on questionnaires, and partly attached copies of their records. Of 1039 couples, 507 met our criteria for lymphocyte immunotherapy (LIT) which they underwent within 2 months after basic evaluation. At LIT and other occasions of personal contact, information on their past history was confirmed, and they were interviewed on daily nicotine consumption. The concept of the study was approved by the ethics committee of the medical faculty of Kiel University and by the local data protection official.

Information on all referred couples constituted the basis of the present evaluation. Missing or abnormal diagnostic results did not lead to exclusion from the study. About 2 years later, the couples were contacted concerning details of further pregnancies and deliveries, on reproductive therapies, diagnostic measures and results, operations, as well as chronic diseases and medication of both partners. Those who had been referred from 2006 to 2008 received a questionnaire in April, 2010, groups 2009–2010 in April, 2012, groups 2011–2013 in April, 2015, and group 2014 in September, 2015. Couples who had not responded were contacted by phone after 3 months. Finally, questionnaires of couples who could not be contacted were sent to the practitioners about 6 months later. Data collection was finalized in July, 2016 comprising 930 couples, and 719 were available for analysis.

The interviews revealed incidentally that three couples separated without children, and four women decided for contraception for personal reasons (*n* = 1), breast cancer (*n* = 2) and intracerebral tumour (*n* = 1). Three couples underwent oocyte donation successfully in another country (as this treatment is prohibited in Germany). They were not excluded from the cohort.

### Variables and main outcome measures

The evaluation was mainly based on questionnaires, and the subgroup who underwent LIT was additionally interviewed before treatment. The study period started at basic evaluation or at LIT. To strengthen validity of the information, the questionnaire sent at the end of the study period was redundant concerning diagnostic measures (laboratory, genetics, laparoscopy, hysteroscopy, sperm count), infertility treatments and all pregnancies which ever had occurred. In case of missing data at first referral, the information obtained later was inserted. In case of discrepancy, the information at first referral was chosen. Main outcome measures were the first pregnancy within the study period and the first pregnancy which lead to delivery. In case of first trimester miscarriage, the date of failure was registered, in case of late miscarriage or delivery, the third gestational month was noted. In eleven cases, a biochemical but no clinical pregnancy was achieved. Overall, this did not change the results; therefore biochemical losses were included as pregnancies. The following details of the past history were introduced at baseline evaluation as possible predictors for outcome: female age, body mass index (BMI), number, gestational age, vital signs, and mode of conception of preceding miscarriages, smoking (in those who were interviewed in the outpatient’s department in the context of LIT), sperm count (classified abnormal at any degree of astheno-teratozoospermia), tubal impairment (preceding extrauterine pregnancies or abnormalities on pelviscopy or hysterosalpingography), and infertility treatment after baseline evaluation as a secondary event. Infertility treatment comprised the methods of autologous artificial reproductive technologies [ART: in vitro fertilisation (IVF)] and intrauterine insemination with the husband’s sperm (AIH). Spontaneous conception included conception after hormonal treatment without further assistance. The definition of idiopathic recurrent miscarriages was based on absence of uterine septae, submucous or multiple uterine fibroids, myomectomy, and intrauterine adhesions (all irrespective of surgical correction), diabetes, gestational diabetes, elevated insulin resistance, cytogenetic abnormalities of either partner, homo- or heterozygous mutations of prothrombin (FII) and factor V (FV), Protein S deficiency, antiphospholipid syndrome, and autoimmune thyroid disease.

### Confounding factors

Application of LIT improved adherence to our centre in terms of current addresses and feedback. Since it was recommended to couples with idiopathic clinical first trimester RPL and no marked infertility, those who received LIT were preselected. When the cohort was categorized for mode of feedback, those returning the questionnaires were most successful, those who were willing to answer on the phone were less successful, and those who could not be contacted personally were least successful according to their practitioners. Statistical chi^2^ analysis revealed that the confounding factors (LIT, mode of feedback, infertility treatment after baseline) each were influenced by female age. In a multivariate approach, these factors were associated significantly with outcome with the exception of LIT (data not shown here). Therefore, we conclude that LIT apparently did not significantly contort our results on prognosis.

### Classification of miscarriages

Preclinical losses were defined as human chorionic gonadotropine G (HCG) positive pregnancies in the 4th to 5th gestational week which were not detected in the uterus by ultrasound (“biochemical pregnancies”). Pregnancies of unknown location were included. Clinical first trimester miscarriages were pregnancies which were located in the uterus by ultrasound and failed between weeks 6 and 12 after the last menstrual period. Losses which occurred from the 13th to 23rd gestational week were termed late miscarriages. For Kaplan–Meier estimation, the time from baseline to gestational week of first trimester miscarriage or to the third gestational month in case of ongoing pregnancy constituted pregnancy rates, and delivery rates accordingly.

### Statistics

**Fig. 2:**
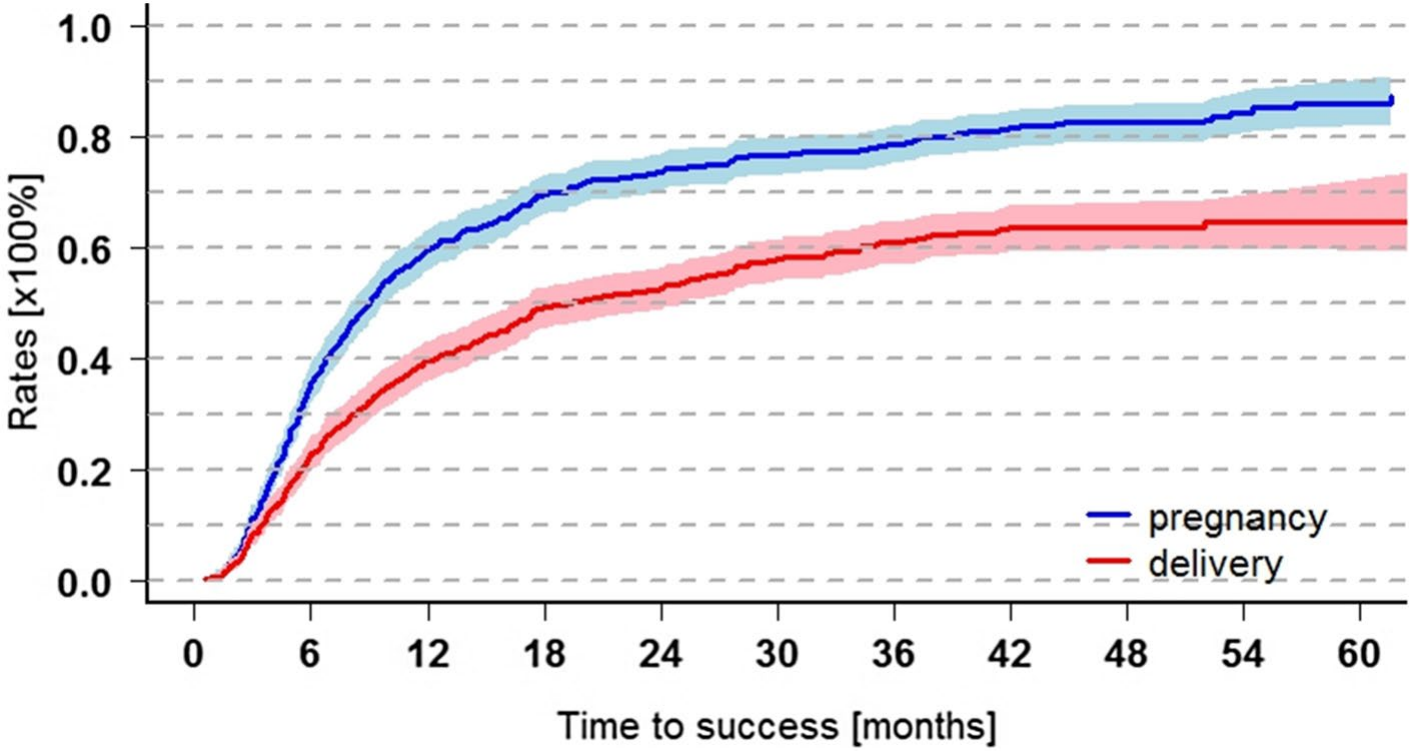
Cumulative pregnancy/delivery rates within 5 years after RPL (*n* = 719), delivery rate: rate of pregnancies which ended in delivery from the 24th gestational week on. Two-year pregnancy rate 73.6 ± 1.7% (95% CI 70.0–76.7%), delivery rate 52.6 ± 1.9% (95% CI 48.7–56.2%). Five-year pregnancy rate 86.1 ± 2.0% (95% CI 81.7–89.5%), delivery rate 64.5 ± 2.2% (95% CI 60.0–68.5%)

**Fig. 3:**
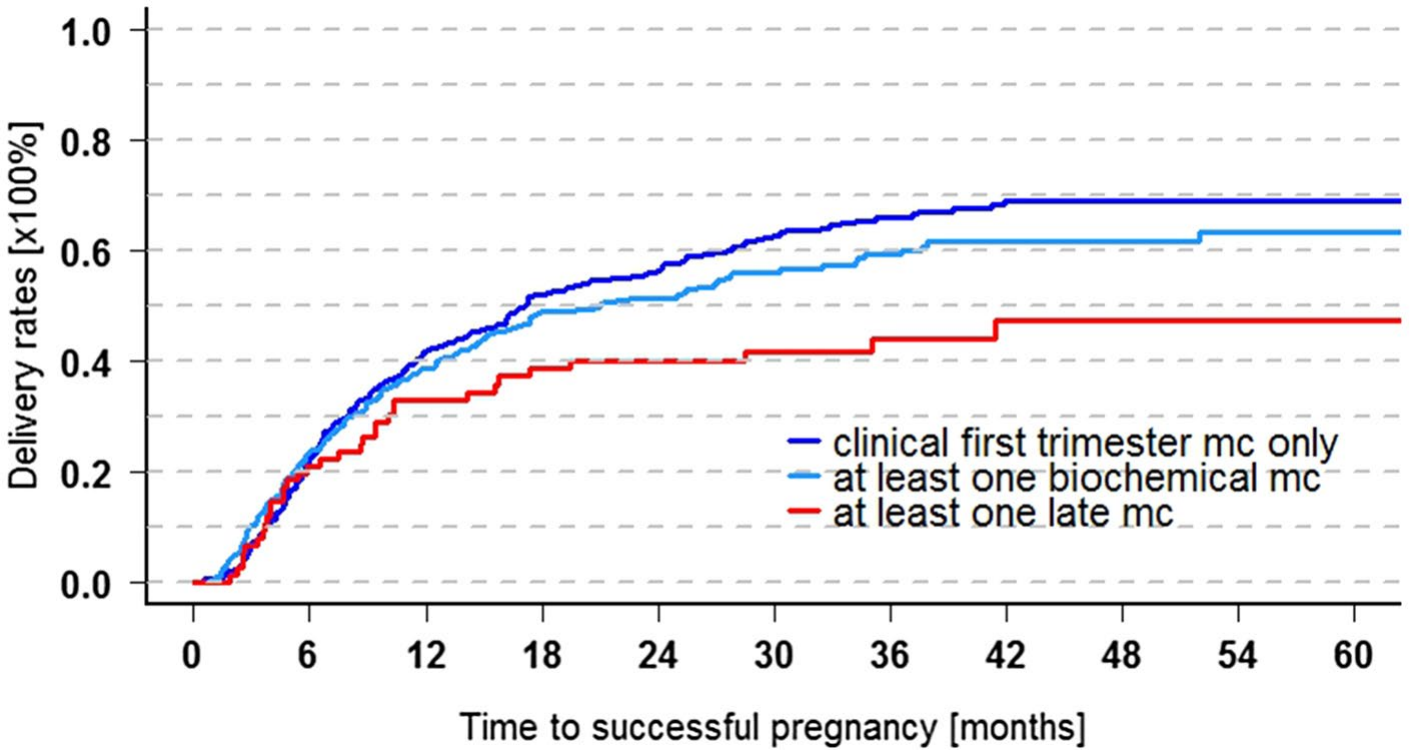
Cumulative 5-year delivery rates with respect to gestational age of baseline miscarriages (*n* = 719). Clinical first trimester PL only (reference): 69.1 ± 2.8% (95% CI 63.0–74.2%, *n* = 344), at least one biochemical pregnancy: 63.3 ± 3.6% (95% CI 55.6–69.7%, *n* = 298, n.s), at least one late PL: 47.3 ± 6.5% (95% CI 32.9–58.5%, *n* = 77, *p* = 0.032). 29 couples had biochemical as well as late PL (mc) and were assigned to the late PL group. Data are shown for the whole cohort, but the result also applied to the fertile subgroup who had got pregnant spontaneously only until baseline

**Fig. 4:**
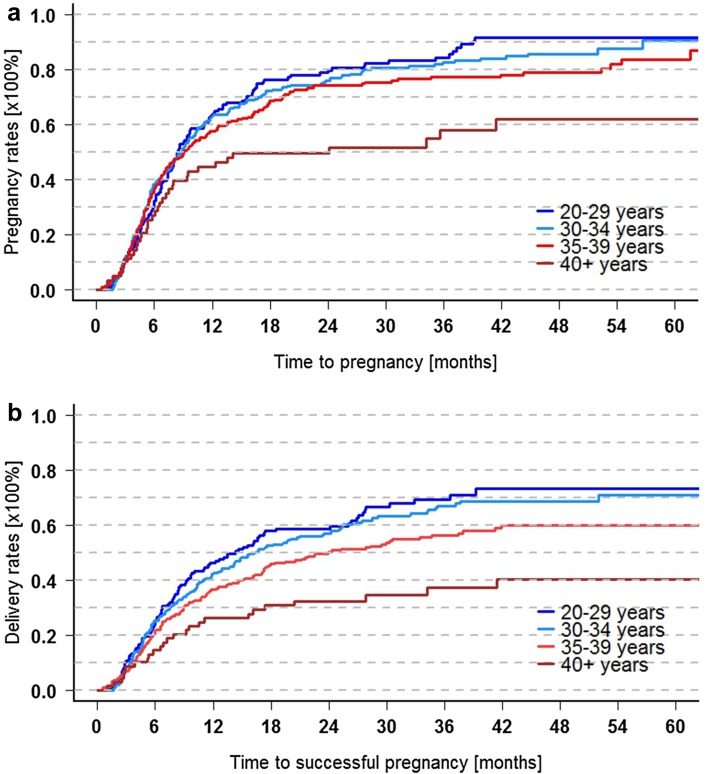
Cumulative (a) pregnancy and (b) delivery rates after RPL, related to female age at baseline (*n* = 719). 20–29 years: *n* = 146, 30–34 years: *n* = 255, 35–39 years: *n* = 248, 40 + years: *n* = 70). Two/five-year delivery rate for women aged 30–34 years: waiting time up to 3 years: 66.8 ± 3.9% (CI 58.3–73.6%)/79.1 ± 3.7% (CI 70.4–85.3%). Waiting time > 3 years: 42.8 ± 5.2% (CI 31.7–52.1%)/53.7 ± 5.6% (CI 41.3–63.6%)

Statistical evaluation was performed using SPSS (SPSS statistics for Windows V22, IBM Corp., Armonk, NY, USA) and the R program (R: A language and environment for statistical computing, R Foundation for Statistical Computing, Vienna, Austria) [24–26]. Most parameters did not follow a normal distribution (Kolmogorov–Smirnov test). Therefore, nonparametric methods were used for data analyses. For comparison of baseline characteristics between ART/AIH and spontaneously conceiving groups (Table 1), the Wilcoxon rank sum test (*U* test) and the Fisher exact test were used. Time to pregnancy and time until birth were calculated from 2006 to July, 2016. Estimated curves for pregnancy and delivery rates (incl. 95% confidence intervals, CI) were constructed by the Kaplan–Meier method. For univariate analyses, baseline variables were correlated with outcome measures using the log-rank test. Those baseline variables which correlated significantly or were of special interest were included into a multivariate analysis for evaluation of their prognostic relevance by applying the Cox proportional hazards model using stepwise backward selection. Hazard or odd’s ratios (OR) were used to describe the 5-year probability of pregnancy or delivery within a subgroup, as compared to its reference group. Probabilities (*p* values) of ≤ 0.05 were considered statistically significant. Univariate and multivariate analysis led to comparable results and levels of significance, irrespective of whether applied to the whole group or to the fertile subgroup who had no ART/AIH but spontaneous conceptions only until baseline. Tables 4, 5 display the multivariate results of this fertile subgroup, whereas Tables 1, 2, 3 (univariate analysis), and 6, Figs. 2, 3, 4a, b are derived from the whole group.

**Table 1:** Baseline characteristics at recruitment (past reproductive history)

Available for analysis	No ART/AIH at baseline	ART/AIH at baseline	*p* value	All
Number of couples	572	147	n.a.	719
Female age at first consultation (in years, median, range)	33.9 (19.7–45.1)	35.8 (25.1–47.8)	< 0.001	34.3 (19.9–47.8)
Age of male partner at first consultation (years, median, range)	35.5 (21.9–61.6)	37.9 (26.8–54.4)	< 0.001	36.0 (21.9–61.6)
Median duration of infertility (years)	3 (1–17)	5 (1–15)	< 0.001	3 (1–17)
Body mass index (in kg/m^2^, mean, range)	23.4 (16.6–43.4)	23.0 (17.2–42.5)	n.s.	23.4 (16.6–43.4)
Daily nicotine abuse present (women)	54/306 (17.6%)	9/82 (11.0%)	n.s.	63/388 (16.2%)
Daily nicotine abuse present (men)	91/303 (30.3%)	15/82 (18.3%)	0.035	106/385 (27.5%)
Idiopathic recurrent miscarriages	412 (72.0%)	109 (74.1)	n.s.	521 (72.5%)
Tubal competence				
Evaluated	184 (32.1%)	88 (59.9%)	< 0.001	272 (37.8%)
Impaired	86 (15.2%)	40 (27.2%)	< 0.001	126 (17.5%)
Male fertility				
Evaluated	381 (66.6%)	142 (98.0%)	< 0.001	487 (67.7%)
Impaired	131 (22.9%)	96 (65.3%)	< 0.001	227 (31.6%)
Previous miscarriages				
Three	385 (67.3%)	106 (72.1%)	n.s.	491 (68.3%)
Four	127 (22.2%)	27 (18.4%)	n.s.	154 (21.4%)
five–nine	60 (15.6%)	14 (9.5%)	n.s.	74 (10.3%)
Number of miscarriages	1999	501	n.a.	2500
Median number per couple (range)	3 (3-9)	3 (3-8)	n.s.	3 (3-9)
Biochemical/preclinical	323 (16.2%)	169 (33.7%)	< 0.001	492 (19.7%)
Embryonic phase (6–12th week)	1528 (76.4%)	297 (59.3%)	< 0.001	1825 (73.0%)
Early foetal phase (13–23rd week)	77(3.9%)	14 (2.8%)	n.s.	92 (3.7%)
Details unknown	62 (3.1%)	21 (4.2%)	n.s.	83 (3.3%)
Mode of conception (at least 1 PL) by				
Spontaneous conception	494	n.a.	n.a.	494 (68.7%)
Hormone stimulation	78	n.a.		78 (10.8%)
AIH	n.a.	37		37 (5.2%)
IVF	n.a	110		110 (15.3%)
Obstetric history				
Preclinical PL present	221 (38.4%)	106 (72.1%)	< 0.001	327 (45.5%)
Women with preclinical PL only	16 (2.8%)	16 (10.9%)	< 0.001	32 (4.5%)
Late miscarriages present	67 (11.7%)	10 (6.8%)	n.s.	77 (10.7%)
Extrauterine pregnancies	55 (9.6%)	11 (7.5%)	n.s.	66 (9.2%)
Deliveries in previous partnership	23 (4.0%)	7 (4.8%)	n.s.	30 (4.2%)
PL in previous partnership	12 (2.1%)	2 (1.4%)	n.s.	14 (1.9%)

The proportion of ART/AIH increased with female age: 11.0% (age 20–29 years, 16/146 couples), 17.3% (30–34 years, 44/255 couples), 27.0% (35–39 years, 67/248 couples), 28.9% (40 + years, 20/70 couples)

*PL* pregnancy loss/miscarriage, *AIH* insemination with husband`s sperm, *ART* in vitro fertilisation (IVF) with/without intracytoplasmic sperm injection (ICSI), *n.a.* not applicable, *n.s.* not significant

**Table 2:** Associated findings in recurrent pregnancy losses (RPL)

719 couples (100%)	No information available	Not evaluated/not performed	Tested-normal	Abnormal^a^
Cytogenetic evaluation				
of female patient	19 (2.7%)	79 (11.0%)	603 (83.9%)	17 (2.4%)^a^
of male partner	20 (2.9%)	101(14.0%)	592 (82.3%)	6 (0.8%)^a^
of at least one preceding miscarriage^b^	19 (2.7%)	522 (72.6%)	95 (13.2%)	83 (11.5%)
Glucose metabolism	18 (2.5%)	458 (63.7%)	213 (29.6%)	30 (4.2%)^a^
Autoimmune thyroid disease^c^	9 (1.2%)	0	652 (90.7%)	58 (8.1%)^a^
Antiphospholipid antibodies (aPL)	39 (5.4%)	241 (33.5%)	432 (60.1%)	7 (1.0%)^a^
Hereditary FII, FV, Protein S deficiency any coagulation abnormality^d^	22 (3.0%)	160 (22.3%)	511 (70.1%)	26 (3.6%)^a^
	As above	As above	353 (49.1%)	184 (25.6%)
Uterine abnormalities (all), in detail:	16 (2.2%)	0	624 (86.5%)	81 (11.3%)
Septum				32 (4.5%)^a^
Unicornuate/bicornuate uterus				9 (1.3%)
Submucous/multiple fibroids, myomectomy				29 (4.0%)^a^
Adhesions/asherman				9 (1.3%)^a^
Myoma regarded irrelevant (subserous, single small fibroids)				9 (1.3%)
Uterine operations^e^	235 (32.7%)	Not performed 32 (4.5%)	n.a.	Performed 452 (62.9%)

^a^Parameters used to define idiopathic recurrent miscarriages, criteria fulfilled: 521 couples (72.5%)

^b^Sex of normal karyotypes mostly unknown

^c^Hashimoto’s disease (*n* = 54), Grave’s disease (*n* = 4), on l-thyroxine: 249 (34.6%)

^d^Additional parameters: MTHFR, Protein C, Antithrombin III, Lipoproteinase a, sticky platelet syndrome, Protein Z

^e^Curettage after miscarriage (429 women, 59.7%), others: conisation, caesarean section, dissection of septum, of adhesions, hysteroscopy

**Table 3:** Cumulative 5-year pregnancy and delivery rates: Univariate analysis (median times and log-rank statistics of whole cohort, *n* = 719)

Main outcome measures	Pregnancy achieved		Delivery achieved		Further PL only
Parameters at basic assessment	No. participants	OR^p^ (95% CI) Rate in %	No. participants	OR^d^ (95%-KI) Rate in %	(OR^p^–OR^d^)/OR^p^(in %)
**Female age (years)**					
20–29	137	92 (84–96)	144	73 (63–81)	20.7
30–34	248	91 (81–96)	253	71 (63–78)	22.0
35–39	238	84 (76–89)	246	60 (52–66)	28.6
40+	63	62 (45–74)	69	40 (26–52)	35.5
All	686	*p* = 0.001	712	*p* < 0.001	
**Body mass index (BMI) in kg/m** ^**2**^					
< 21	159	86 (78–92)	164	69 (59–76)	19.8
21–24	272	91 (83–96)	279	69 (62–75)	24.2
25–29	165	80 (71–85)	174	58 (49–66)	27.5
30+	66	77 (60–87)	69	60 (25–78)	22.1
All	662	*p* = 0.057 n.s.	686	*p* = 0.014	
**Waiting time/period of infertility (years)**					
0–3	377	91 (86–94)	389	76 (70–80)	16.5
> 3	295	81 (71–88)	310	50 (43–56)	38.3
All	672	p < 0.001	699	p < 0.001	
**Clinical 1st trimester miscarriages**					
3	303	86 (80–91)	312	68 (61–73)	20.9
4	98	85 (73–91)	106	63 (50–72)	25.9
5–9	41	84 (63–93)	42	57 (37–71)	32.1
All	442	*p* = 0.570 n.s.	460	*p* = 0.195 n.s.	
**Late miscarriages present (13th–23rd gestational week)**					
No	616	87 (82–90)	637	67 (62–71)	23.0
Yes	70	77 (62–87)	75	46 (32–58)	40.3
All	686	*p* = 0.079 n.s.	712	*p* = 0.008	
**Mode of conception in preceding miscarriages (assisted conception at least once)**					
Spontaneous/ hormonal treatment	545	87 (82–90)	565	65 (60–70)	25.3
AIH/ IVF embryo transfer	141	84 (71–91)	147	63 (53–71)	25.0
All	686	*p* = 0.157 n.s.	712	*p* = 0.273 n.s.	
**Nicotine abuse**					
No	320	91 (83–95)	325	71 (65–76)	22.0
Yes	63	87 (69–95)	63	68 (49–80)	21.8
All	383	*p* = 0.064 n.s.	388	*p* = 0.429 n.s.	
**Evaluation of tubal competence**					
Not assessed	426	89 (84–92)	438	70 (64–75)	21.8
Results normal	141	84 (71–91)	146	62 (51- 70)	26.2
Results abnormal	117	75 (65–83)	126	50 (40–59)	33.3
All	684	*p* = 0.003	710	*p* = 0.001	
**Evaluation of sperm quality**					
Not assessed	175	85 (76–91)	185	68 (59– 75)	20.0
Results normal	284	90 (94–82)	294	68 (61–73)	24.4
Results abnormal	221	81 (72–87)	227	56 (48–63)	30.9
All	680	*p* = 0.019	760	*p* = 0.002	
**Criteria for idiopathic PRL fulfilled**					
No	181	82 (75–88)	194	64 (55–70)	22.0
Yes	505	88 (82–92)	518	65 (60–70)	26.1
All	686	*p* = 0.864 n.s.	719	*p* = 0.868 n.s.	

*CI* confidence interval,* OR* mean likelyhood (odd´s ratio), * OR* ^p^ mean pregnancy rate,* OR* ^d^ mean delivery rate

## Results

### Study group (Table 1)

Of 719 available couples, 400 (55.6%) returned the questionnaire, 173 (24.1%) were contacted by phone only, and in 146 cases (20.3%) information was given by the practitioners only.

At baseline assessment, the women were 34.1 (19.7–47.8) years, their male partners 36.5 (21.9–61.6) years old. Female body mass index was 24.3 (16.1–43.4) kg/m^2^ (693 women informative), 63 of 388 women (16.2%) undergoing LIT were smokers (Table 1).

Within a median waiting time of 3 (1–17) years they had a median of 3 (3–9) miscarriages. Most of these (1825/2500, 73.0%) were first trimester clinical losses. Vital signs were reported positive in 731 (40.1%) of them, and were unknown in 104 (5.7%). No clear information was available as to whether vital signs had ever been present in pregnancy or in the final stage before the miscarriage was diagnosed. The majority (572 couples, 79.6%) had conceived spontaneously or at least once after hormonal supportive therapy, while 147 (20.4%) had been pregnant at least once after AIH or IVF. Report on vital signs did not differ between these subgroups (616/1528 = 40.3 vs. 115/297 = 38.7% of first trimester miscarriages). The couples under ART/AIH were significantly elder and had longer waiting times; especially male factor subfertility was much more prevalent than in those without ART/AIH. The proportion of preclinical losses was twice as high in the ART/AIH group and increased with female age.

### Main outcome (Fig. 2)

After a median follow-up of 33.7 months (range 1.7–123.9 months), 686 of 719 couples were informative concerning pregnancy and 547 got pregnant. The other 33 women indicated that they did not conceive but it is unknown whether they had further miscarriages. Concerning delivery, 712 of 719 couples were informative, and 417 were delivered, while in 7 cases, the outcome of pregnancy was unknown.

Using Kaplan–Meier estimation, within 5 years 86.1 ± 2.0% (81.7–89.5%) got pregnant, followed by delivery in 64.5 ± 2.2% (60.0–68.5%). Thus, within 5 years, about 20.1% had further miscarriages only, and 13.9% did not get pregnant again. Two-year pregnancy rate was 73.6 ± 1.7% (70.0–76.7%), delivery rate 52.6 ± 1.9% (48.7–56.2%).

### Prognosis after preclinical losses and late miscarriages (Fig. 3)

At baseline, nearly half of the cohort (327 of 719, 45.5%) had experienced one or several biochemical pregnancies (range 1–5 losses), and 77 women had suffered foetal losses (10.9%. range 1–4 losses) (Table 1). For Kaplan–Meier evaluation, those 29 women who had a mixture of biochemical and late miscarriages were assigned to the late miscarriage group.

One half (344 couples, 47.8%) had neither of these but clinical early (embryonic) miscarriages only. Kaplan–Meier evaluation revealed that they had the most favourable prognosis. Preceding preclinical losses indicated a trend towards reduced fertility (n.s.), whereas after late miscarriages, the chances for delivery were significantly reduced (*p* = 0.032). For univariate analysis, therefore we did not differentiate between clinical or preclinical early miscarriages.

### Associated findings and recurrent miscarriages of unknown cause (Table 2)

In 198 (27.5%), relevant pathologies had been identified according to the guidelines; the majority of 521 women (72.5%) had idiopathic miscarriages. An antiphospholipid syndrome was only rarely described. The panels of tests for coagulation defects varied but had been performed in nearly 75% of the women. Uterine evacuation (times 1–9) had been reported by nearly 60% of the women; which surgical methods had been used is not known. Three quarters of the women were on l-thyroxine, some because of hypothyroid disease, mostly to reach an ideal TSH level.

### Univariate analysis of infertility and lifestyle variables (Table 3, Fig. 4a, b)

Several variables were each tested for their statistical impact on pregnancy and delivery rates. The median values (odd’s ratios) and confidence intervals represent the pregnancy and delivery rates of the respective subgroups and are customized in “%”.

Increasing female age (Fig. 4a, b), tubal and sperm impairment as well as waiting time over 3 years were significantly associated with lower pregnancy and delivery rates. Prospects after waiting time did not differ when it had been shorter than 3 years, but declined markedly from the 4th year on (evaluation of years 1–3 not shown). Being overweighed (BMI exceeding 24 and 29 kg/m^2^) was associated with further miscarriage, a trend to reduced pregnancy rates and a significant reduction in delivery rates. This also applied to couples who had experienced a late miscarriage.

Evaluation did not provide significant associations with number of preceding miscarriages, mode of conception until baseline (non-ART/AIH or ART/AIH), classification into non-idiopathic and idiopathic miscarriages, and nicotine abuse in our collective.

There was a trend towards reduced fertility after five and more clinical miscarriages, but the chances did not differ markedly after three or after four clinical miscarriages. The group of couples with five and more miscarriages was small, comprising 6% of the whole cohort (42/712 couples). Smoking habits of the women showed a non-significant negative trend on pregnancy but not delivery rates. Moreover, nicotine abuse and age of the male partner did not significantly relate to sperm quality and outcome measures (data not shown).

Apart from a difference between couples who had or had not experienced late miscarriages, we were unable to show that the gestational stage of development in preceding clinical miscarriages was meaningful with respect to outcome. Neither the report on vital signs nor mean gestational week of the first three miscarriages, nor of the first three clinical early miscarriages showed a significant relationship with outcome.

As mentioned above, prognosis was not different after idiopathic (unexplained) or non-idiopathic recurrent miscarriages. Of note, the pathologies of those classified as non-idiopathic were partly corrected by surgical or pharmaceutical measures before or during the observational period.

Although our evaluation on cumulative miscarriage rates is a rough estimate, our data may suggest that the risk to experience further miscarriages not only rises with female age, waiting time, and number of preceding miscarriages, but also with tubal impairment, and possibly male factor infertility.

### Multivariate analysis of infertility and lifestyle variables (Tables 4, 5)

All parameters which were associated significantly with pregnancy or delivery rates (female age, BMI, waiting time, presence of late miscarriage, tubal and male factor infertility) and classification into idiopathic RPL were included into the multivariate analysis. It was applied to the whole as well as to the fertile subgroup of couples who had spontaneously conceived RPL, and practically identical results were obtained (details not shown). We conclude that parameters related to subfertility significantly relate to outcome, not only in the group who had required ART/AIH to get pregnant but also in those who had conceived spontaneously until baseline.

**Table 4:** Relative chance to get pregnant within 5 years after a history of spontaneously conceived RPL

Parameters at baseline	Participants^a^ 509/572 (89.0%)	OR	95% CI	*p* value
Female age groups (years)				
20–29	114	0.99	0.76–1.27	0.910
30–34	191	1	Reference	
35–39	164	0.84	0.66–1.06	0.137
40+	40	0.58	0.38–0.89	0.013
Waiting time (years)				
0–3	324	1	Reference	
> 3	185	0.69	0.56–0.85	0.001
Evaluation of sperm quality				
Not assessed	164	0.87	0.69–1.09	0.217
Results normal	222	1	Reference	
Results abnormal	123	0.75	0.58–0.96	0.022

Multivariate analysis following stepwise Cox logistic regression model. Eligible: 509 women, pregnancy achieved: 412 women. Further comments: See table 5

^a^63 observations removed because of missing data

**Table 5:** Relative chance of subsequent delivery within 5 years after a history of spontaneously conceived RPL

Parameters at baseline	Participants^a^ 529/572 (92.5%)	OR	95% CI	*p* value
Female age groups (years)				
20–29	120	0.94	0.71–1.23	0.688
30–34	194	1	Reference	
35–39	170	0.74	0.56–0.95	0.029
40+	45	0.42	0.26–0.74	0.001
Waiting time (years)				
0–3	335	1	Reference	
>3	194	0.54	0.41–0.70	< 0.001
Evaluation of tubal competence				
Not assessed	352	1.06	0.79–1.44	0.692
Result normal	95	1	Reference	
Result abnormal	122	0.66	0.43–1.01	0.053
Evaluation of sperm quality				
Not assessed	172	0.91	0.70–1.18	0.463
Results normal	231	1	Reference	
Results abnormal	126	0.67	0.49–0.91	0.009

Multivariate analysis following stepwise Cox logistic regression model. Eligible: 529 women, delivered: 312 women

Parameters included: In contrast to delivery rates, pregnancy rates did not differ statistically when stratified for tubal factors. There was no significant correlation of BMI, late miscarriages, and idiopathic RPL with outcome measures in the multivariate model

^a^43 observations removed because of missing data

Therefore, the results of the non-ART/AIH group are displayed (Tables 4, 5). They show that female age, waiting time (> 3 years), and male factor infertility were significantly associated with reduced pregnancy and delivery rates. The impact of tubal function was significant concerning delivery but not pregnancy rates, suggesting that women with tubal impairment retained a higher risk to miscarry again.

### Pregnancies and deliveries after ART/AIH and spontaneous conception (Table 6)

In 629 of 719 women, there was sufficient information concerning mode of conception after baseline. Whereas 147 of 719 (20.4%) were under ART/AIH at baseline (Table 1), the figure rose to 30.6% (199 of 629 couples) after baseline, while the other 430 couples (69.4%) tried to conceive spontaneously. In the ART/AIH group, 143 of 199 (71.9%) got pregnant, and 106 (53.3%) deliveries were reported. Among the non-ART/AIH couples, 382 of 430 (77.5%) got pregnant spontaneously again after baseline, and 294 (68.4%) were delivered thereafter. The figures indicate that there may not be a clear-cut border between recurrent miscarriages and secondary sterility.

**Table 6:** Characteristics of singleton deliveries, related to mode of conception (*n* = 377)

	Spontaneous conception	ART/AIH
Deliveries	294 of 400	106 of 400
Mode of conception known of these singleton deliveries:	275 of 377 (72.9)	102 of 377 (27.1)
Gender ratio male/female	123/134 (0.9)	51/49 (1.0)
Gender unknown	18 (6.5)	2 (2.0)
Delivery < 32nd week^a^	6 (2.2)	5 (4.9)
Delivery < 37th week^a^	32 (11.6)	13 (12.7)
Term delivery	243 (88.4)	89 (87.3)
Median weight of term infants (in g)^a^	3260	3240
Median gestational week of term infants	39 (37–42)	40 (37–44)
Perinatal death^b^	2 (0.7)	0

Deliveries of the whole cohort: *n* = 417, of these deliveries with known mode of conception: *n* = 400

Ongoing pregnancies, or information on newborn unavailable: *n* = 19, multiple gestation: *n* = 21

^a^Rates of premature and very premature deliveries and weight of term babies did not differ significantly

^b^Intrauterine foetal death at term, postnatal death due to extreme prematurity (two boys)

Of 417 deliveries, 377 children were singletons, and enough information on mode of conception and further details had been obtained (Table 6). When comparing the ART/AIH and non-ART/AIH singletons, neonatal weight and gestational age at delivery did not differ statistically between the two groups.

## Discussion

### Key results

In a large cohort comprising 719 couples, we evaluated 5-year cumulative pregnancy as well as delivery rates after recurrent miscarriages. The data show that recurrent miscarriages mainly represent a form of subfertility also involving the male partner. Among the factors evaluated here, female age and waiting time had a highly significant impact on prognosis. Moreover, other markers of infertility (e.g., tubal dysfunction and reduced sperm count) were more powerful indicators than already well- described variables (as number of preceding miscarriages and female body mass index). There was no difference in outcome, irrespective of whether or not RPL had occurred under ART/AIH at baseline. In comparison to diagnostic variables linked to subfertility, the classification into idiopathic and non-idiopathic recurrent miscarriages seems to be of limited value.

### Subfertility and recurrent miscarriages

It proved difficult to clearly differentiate between couples who had a problem of repeated miscarriages only and those who had additional marked subfertility, as an increasing number (from 20.4% at baseline to 30.6%) entered an ART/AIH programme during the observational period. The border between spontaneously conceiving couples and those who required or decided for ART or AIH appeared to be fluctuant. Our findings on pregnancy rates underline that after miscarriage, there is a trend towards secondary sterility.

The prognosis is most favourable after clinical first trimester miscarriages. Preclinical miscarriages did not only occur frequently after ART/AIH cycles, but were also reported after spontaneous conception (Table 1). Therefore, their detection may not only be the result of increased awareness of couples under infertility treatment who initiate HCG testing early because of intended conception. By themselves preclinical losses may be a marker of reduced fertility, as has been described previously by Kolte et al. [27], and may partly be attributed to extrauterine pregnancy and tubal incompetence.

Late miscarriages have also been linked to reduced fertility [18]. Since the risk of late miscarriages is higher after in vitro fertilisation, as compared to other modes of conception, our result could have been biased. Therefore, we conducted the univariate analysis not only on the whole cohort but also in the subgroup who had not been under ART/AIH at baseline (latter evaluation not shown). The statistical result was the same, ruling out an effect caused by underlying ART/AIH. We cannot exclude that psychological factors play a role in couples experiencing late miscarriages, since such an event may be even more traumatizing than early miscarriages and may prompt prospective parents to decide for contraception.

Waiting time of not more than 3 years until baseline indicated a favourable outcome. Defining shorter periods (e.g., three miscarriages within 1–2 years) did not prove to be more advantageous in our cohort (data not shown). We conclude that also in the context of RPL, fertility is optimal when the interval has been at least one pregnancy per year. This means that the acceptable waiting time is about 6–9 months per pregnancy; a result which has been shown elsewhere concerning time to pregnancy after miscarriage [28] and infertility [29]. In our opinion, this should also be applied to women below 30 years of age.

We assume that tubal function was evaluated in the referring centres only when the time to pregnancy was judged too long. Those who had achieved three or more intrauterine pregnancies within 3 years were unlikely to have been examined at baseline. The fact that a woman was examined at all indicated that some sort of tubal subfertility was suspected even when she was found to have a normal tubal passage. Tubal impairment may prevent successful implantation, e.g., by prolonged embryonic passage to the receptive endometrium, by blockage, or by concomitant endometrial dysfunction. Possibly tubal and endometrial tissue should be regarded as a functional unit which can be disturbed by the same damaging events.

Male subfertility so far is not a part of the general work-up after miscarriages, and practitioners may not generally consider evaluation after the woman has become pregnant repeatedly. Our data suggest that there should be awareness of an andrological factor when the waiting time exceeds about 6-9 months.

Nicotine abuse has been reported to promote infertility and miscarriage [30]. We demonstrated a negative trend only on pregnancy rates (Table 3) which may be explained by the fact that data on nicotine habits were available only in half of the cohort. Moreover, we did not have information on pack years.

### Idiopathic recurrent miscarriages

Retrospectively, we categorized the participants of the study into those who had explained and unexplained recurrent miscarriages, by following the criteria of current guidelines. The rate of parental chromosome abnormalities, diabetes, autoimmune thyroid disease, and uterine abnormalities were in line with figures derived from the general or RPL population [4, 5, 31–33]. Although an overall rate of 72% of unexplained cases is plausible, in detail the rate of women with elevated antiphospholipid antibodies (aPL) in our cohort was lower than expected in RPL [4, 5] but corresponded to the rates reported for aPL and APS in the general population [34]. This may indicate that the prevalence of antiphospholipid syndrome (APS) is overestimated in the RPL population, or it reflects a pre-selection bias. Women with proven APS may not have been referred to a tertiary immunological care centre but were treated locally. Under-reporting of laboratory data may also be involved, as FII and FV mutations were less frequently reported than expected in a general population of mainly Caucasian descent [34].

The analysis showed that the classification in explained or unexplained RPL did not have an impact on prognosis, similar to the findings reported by Bricker and Farquharson [35]. There may be two components to explain this. Either the pathologies are treated very effectively, or the definition of idiopathic RPL is less valuable than proposed. If “explained” RPL are ascribed to a mixture of parameters related to subfertility (e.g., uterine malformation or adhesions, diabetes, and untreated clinically symptomatic thyroid disease), and of parameters which may be not (parental chromosomal aberrations, APS, coagulation defects, normal TSH levels above 2.5 IU/ml), this group will comprise women with an unfavourable as well as a benign prognosis. Their general outcome will depend on the proportion of individuals with these diagnoses within the group. The term “Idiopathic” RPL may be defined as RPL “basically caused by a reduced embryonic capacity to develop”. Such a state cannot be overcome by treating maternal conditions with immunotherapies or other measures.

### Comparison of outcome measures with other studies

Several epidemiological studies have been published in recent years to estimate the delivery rates related to female age, number of previous miscarriages, lifestyle factors like BMI and smoking, and the impact of biochemical pregnancies or pregnancies of unknown location, some of whom are cited [15, 17, 18, 27, 30, 36, 37].

We compared our data to our previous evaluation [21] on a similar cohort and to a recent study of similar size [17].

Our recent evaluation comprised 228 nulliparous women referred from 1996 to 2003. As in the present study, the outcome measures were cumulative pregnancy and delivery rates [21]. As confirmed here, these correlated significantly with waiting time and maternal age, and did not vary markedly in the age groups under 35 years. But obviously, the cohorts of the two studies were different from each other. The proportion of preclinical pregnancies at baseline was higher in the current group who had conceived spontaneously (8.2 vs. 16.2%, see Table 1) than in the previous evaluation, suggesting a lower overall fertility and explaining a less favourable outcome than reported previously (e.g., successful pregnancy rate 84.9 vs. 56.9% within 2 years in age group 30–34 years). This discrepancy may be explained by pre-selection of the practitioners in later years who may have referred more couples with obvious fertility problems. Moreover, the previous evaluation was not based on a Kaplan–Meier estimation. Instead, couples who actually were available after 24 months until delivery were collected so that we might have missed unsuccessful couples. Moreover in the present evaluation, we included couples with preceding late miscarriages who are shown to have a reduced chance to conceive successfully.

As explained above, we were not able to confirm our preliminary result that vital signs in the first miscarriages may have prognostic properties. This hypothesis would need re-evaluation in a gynaecological unit based on medical records. Moreover, we could not verify that the number of previous losses in the preceding miscarriages are correlated significantly with outcome.

Lund et al. evaluated delivery rates in 987 women after three or more idiopathic miscarriages who partly required infertility treatment [17]. They reported an overall live birth rate of 66.7% within 5 years which is in line with our result (64.5%). Median female age was lower (32.1 vs. 34.7 years) in their cohort. Their 5-year results in age groups 30-34 years to 40 + are merely identical to our results (69.9, 60, and 40%, see Table 3), whereas women below 30 years at baseline displayed higher delivery rates of 80%. Possibly in our cohort, couples suffering from marked infertility were over-represented in this youngest age group.

In contrast to the above mentioned [17, 21] and other evaluations, the number of preceding miscarriages did not have a significant influence. As an explanation, Lund et al. had twice as many couples with five and more miscarriages (230/987, 23.3%), as compared to our cohort (74/719, 10.3%, Table 1) leading to a higher statistical impact.

## Strengths and limitations of the study

Immunological evaluation at our centre was appreciated by patients and practitioners as an additional counselling and eventual therapeutic perspective. Therefore, we were able to accumulate details of a large cohort on past obstetric history and diagnostic results in a prospective manner. Since couples were referred from various centres all over Germany irrespective of their socio-economic status, the participants of our study may represent a national cross-sectional cohort. We cannot exclude, though a pre-selection bias concerning associated coagulation defects and APS.

By generating redundancy from patients and practitioners, we took into account that data on outcome were mainly based on questionnaires. Nevertheless, we may not have succeeded in avoiding a reporting bias in certain aspects. In particular, data on the embryonic stage of development and vital signs of miscarriages were difficult to assess. The accuracy of tubal evaluation depended on the diagnostic mode used locally (e.g., ultrasonography, pelviscopy) and the results could not further be differentiated, e.g., concerning possible cause and grade of tubal impairment. In male factor infertility, we did not have information whether the evaluation was done in a certified laboratory following WHO criteria and whether the results were reproducible over time.

Statistical Kaplan–Meier estimations of success rates extrapolate courses of observation into the future (here: 5 years) although the median observational period in the cohort was shorter (nearly 3 years). Their accuracy is dependent on sample size and may have been lower in small subgroups.

## Conclusion

Miscarriages can be a traumatising experience for couples who long to have children. After repeated miscarriages, the concept of looking for presumptively underlying maternal conditions may further undermine self-confidence of the prospective mother. In an effort to help, it can lead to therapeutic proposals which are prescribed off-label to women who usually still have good perspectives to conceive a child. A possible teratogenic effect of these therapies is neglected.

If miscarriages were interpreted mainly as a form of subfertility instead, preventive strategies which preserve fertility of both partners, counselling on their chances to conceive, and psychological support (“tender loving care” [38]) would be valued as primary elements of medical care from a first miscarriage on.

In our experience, lymphocyte immunotherapy has been accepted well by RPL couples as an optional treatment for decades. Among others, one possible reason for their adherence was that LIT defined RPL as a shared problem of both partners. The rationale for immunotherapies in general and LIT in particular has some historical dimension which is not discussed here.

Present knowledge indicates that LIT as well as other concepts for treating women with unexplained RPL should be replaced by a preventive approach, as outlined above. Nevertheless, integrating the partner into the diagnostic process helps to give emotional support to the woman and is an aspect which should and can be retained well in our opinion.

The group of couples experiencing RPL may be less heterogeneous in terms of underlying associated factors (“causes”) but mainly with respect to underlying fertility. It does not only decline with maternal age but also diverges within age groups [29]. RPL couples can be viewed at as a group who do not display optimal fecundity but retain similar probabilities of conceiving a child as couples who have not conceived at all within 1 year [39].

We suggest to implement basic medical care after any miscarriage before RPL have actually occurred. Surgical evacuation should be avoided after early clinical miscarriage as best as possible [40, 41]. If evacuation is necessary genetic evaluation of embryonic tissue is an option to reveal embryonic causes of this particular miscarriage. Underlying subfertility should be considered if the observed time to pregnancy exceeds 6–9 months-also in women below 30 years. The first step of prevention could be education on behaviour and lifestyle variables (e.g., role of moderate physical training, body weight/BMI > 29 kg/m^2^, alcohol and nicotine abuse of both partners) [37]. In a second step, both partners could be evaluated for female and male factor infertility, and infertility treatment discussed according to the results. Couples should be informed that miscarriage can occur again at a somewhat higher rate than in the general population but chances to conceive a child are generally retained.

In some constellations, appropriate solutions may require further research and clinical approaches which are beyond the scope of this evaluation, e.g., for couples with second trimester miscarriages and for those who experience high grade recurrent first trimester clinical losses within short times to pregnancy.
